# Orthogonal Frequency Division Multiplexing Techniques Comparison for Underwater Optical Wireless Communication Systems

**DOI:** 10.3390/s19010160

**Published:** 2019-01-04

**Authors:** Jie Lian, Yan Gao, Peng Wu, Dianbin Lian

**Affiliations:** 1 Department of Electrical and Computer Engineering, University of Virginia, Charlottesville, VA 22903, USA; 2 Information Engineering School, Xi’an University, Xi’an 710065, China; Chouyanchouyan0824@gmail.com; 3 Xi’an Modern Control Technology Research Institute, Xi’an 710065, China; wupengrock@163.com; 4 Electronics and Information School, Northwestern Polytechnical University, Xi’an 710072, China

**Keywords:** optical wireless communications, underwater communications, OFDM, peak power constraint, propagation distance, BER, band-limited channel, turbulence fading

## Abstract

Optical wireless communication is an energy-efficient and cost-effective solution for high-speed and highly-secure wireless connections. In this paper, we compare, discuss, and analyze three popular optical orthogonal frequency division multiplexing (OFDM) techniques, such as DC-biased optical OFDM (DCO-OFDM), asymmetrically-clipped optical OFDM (ACO-OFDM), and unipolar OFDM (U-OFDM), for underwater optical wireless communication systems. The peak power constraint, bandwidth limit of the light source, turbulence fading underwater channel, and the channel estimation error are taken into account. To maximize the achievable data propagation distance, we propose to optimize the modulation index that controls the signal magnitude, and a bitloading algorithm is applied. This optimization process trades off the clipping distortion caused by the peak power constraint and the signal to noise ratio (SNR). The SNR and clipping effects of the three compared OFDM techniques are modeled in this paper. From the numerical results, DCO-OFDM outperforms ACO- and U-OFDM when the transmitted bit rate is high compared to the channel bandwidth. Otherwise, U-OFDM can provide a longer propagation distance or requires less transmitted power.

## 1. Introduction

In recent years, underwater wireless communications (UWC) has attracted more and more research attention due to the increasing civilian and military demands [1,2,3]. For example, underwater vehicles need high-speed and long-distance wireless connections between each other. The underwater sensor network needs a reliable link to transmit the collected data back to the ground. Radio-frequency (RF) waves, acoustic waves, and optical waves are three possible transmission media for UWC. Due to the high security and transmission speed, underwater optical wireless communication (UOWC) becomes a more and more promising technique, supporting highly-reliable wireless underwater connections [4].

Compared with optical wireless communications, underwater acoustic communication has a longer application history due to its low attenuation level and long propagation distance [1]. However, the slow data rate and severe delay are the drawbacks for underwater acoustic systems. Although RF communication can provide a much higher data rate than acoustic systems, the extremely limited propagation range due to the high attenuation of radio waves in the water limits its application. UOWC is an emerging technique for underwater systems with a high-speed transmission for moderate distance communication. Although UOWC can provide a longer propagation distance than underwater RF systems, an extremely long-range underwater communication should currently rely on acoustic systems. Therefore, in UOWC, most of the research focuses on improving the transmission data rate and propagation distance [5,6,7].

A light-emitting diode (LED) working as a high power-efficient light source has attracted more attention recently and shows a big potential in underwater optical wireless communication systems [8,9,10,11]. Single-carrier frequency division multiple access (SC-FDMA) can achieve a lower peak to average power ratio than OFDM, which has the potential to be beneficial for underwater optical wireless communications as well [12,13,14,15]. Since LED is a band-limited device, multi-carrier modulation is more suitable for the LED-based UOWC systems. Orthogonal frequency division multiplexing (OFDM) is the key technique for 4G wireless communications, which is also widely used in many applications. Due to its resistance to inter-symbol interference (ISI) and high spectral efficiency, OFDM is a candidate technique for optical wireless communications. In OFDM techniques, *M*-ary quadrature amplitude modulation (M-QAM) is commonly used for a high efficiency of bandwidth. Unlike the conventional RF OFDM, the OFDM for the optical wireless communication systems can only transmit real and non-negative signals. Nowadays, optical OFDM has attacked much attention, both in academia and industry [16,17,18]. Recently, there have been many popular optical OFDM techniques such as DC-biased optical OFDM (DCO-OFDM), asymmetrically-clipped optical OFDM (ACO-OFDM), and unipolar OFDM (U-OFDM) [19,20,21]. To avoid the negative signals, DCO-OFDM adds a DC bias to the original bipolar real-valued OFDM signals. However, since the DC-biased signals cannot totally get rid of negative-valued information, the signals under zero must be hard clipped. ACO-OFDM and U-OFDM are recently-proposed optical OFDM techniques for intensity modulation and direct detection systems [20]. For ACO-OFDM, only odd frequency subcarriers are used for modulating data, and even frequency subcarriers are set to zero. By that, the zero clipping effects can only introduce interference on the even subcarriers [22]. In U-OFDM, the values of positive and negative information are transmitted successively; therefore, there is no zero clipping distortion.

Underwater optical OFDM has attracted much research attention recently since OFDM is a candidate technique to provide high-speed transmission. Some related work based on underwater optical OFDM has been proposed [11,23,24]. The researchers presented an experimental demonstration to show that the underwater OFDM can support up to a 15.36-Mbps data rate for transmitting 4 m when the bandwidth of the LED is 10 MHz [11]. Using a a two-stage injection-locked 405-nm blue light laser diode transmitter, a data rate can be achieved of several gigabits per second [24].

Since the light sources for optical wireless communications are nonlinear and power limited, both the zero and peak power clipping should be considered. In this paper, we compare, analyze, and discuss both the zero and peak power limit distortions for DCO-, ACO-, and U-OFDM in the applications of underwater optical wireless communication systems. The propagation distance, bit error rate (BER), and data throughput are used as evaluation criteria in this paper.

The contributions of this paper are listed below:

- A comparison of the state-of-the-art optical OFDM techniques for underwater optical wireless communication systems is discussed based on the propagation distance, BER, and data throughput.
- Considering the turbulence fading underwater channel, the performances of ACO-, DCO-, and U-OFDM using the channels with different bandwidths are tested.
- For different transmitted bit rates, the achievable propagation distances of DCO-, ACO-, and U-OFDM with the band-limited channel are discussed.
- The underwater channel uncertainty caused by the pointing error is modeled and tested.

The notations in this paper are summarized and shown in Table 1, and the rest of the paper is organized as follows. The research problem is described in Section 2. System models and performance analysis are described in Section 3. Section 4 considers the band-limited channel and discusses the system performance. In Section 5, numerical results are shown. Finally, conclusion and future work are discussed in Section 6.

## 2. Research Problem

This section describes the differences between RF and optical OFDM, the underwater optical wireless communications channel model, and the impacts of the power and bandwidth limitations of LEDs.

### 2.1. RF OFDM vs. Optical OFDM

OFDM is a popular technique that is designed for RF systems due to its robustness to intersymbol interference and frequency selective channels [25]. The optical OFDM works as a candidate for high-speed transmission and has attracted more and more attention [26]. However, the conventional RF OFDM cannot be directly adopted in optical systems. For RF systems, both negative and positive complex-valued signals can be transmitted. Unlike the RF systems, optical wireless communication systems can only generate non-negative and real-valued signals. Therefore, Hermitian symmetry is usually used to generate real-valued signals [22]. Compared with RF OFDM, the bandwidth efficiency of optical OFDM is reduced by half due to the Hermitian symmetry.

### 2.2. Underwater Optical Channel Model

Due to the unique physical characteristics of underwater light propagation, absorption and scattering are the two major factors that impact the channel loss. The absorption and scattering coefficients highly depend on the water types. The work in [27] investigated the attenuation coefficients for different water types, such as pure sea, clear ocean, coastal, and harbor. In Table 2, some typical attenuation coefficients are illustrated. From the results in this table, the pure sea water has the smallest absorption and scattering coefficients, which result in the least signal attenuation.

**Figure 1:**
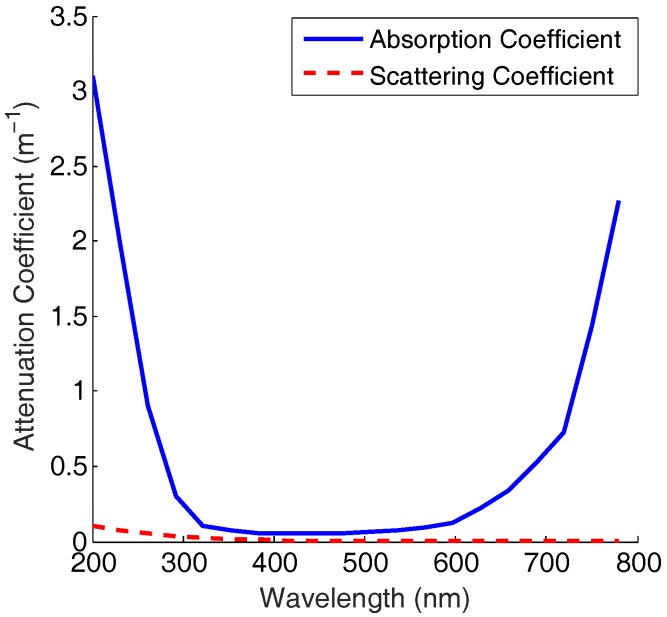
Absorbing and scattering coefficients for different wavelengths in pure sea water [28].

Light with different wavelengths experience different signal attenuation. Figure 1 shows the attenuation coefficient in pure sea water [28].

For different light wavelengths and water conditions (deep water or harbor area), the absorption and scattering may vary. In this paper, the effects of water conditions are beyond the scope; therefore, the effect of light wavelength is considered only. In UOWC, the overall attenuation of absorption and scattering can be described as [4]:

(1) c(λ)=a(λ)+b(λ),

where a(λ) and b(λ) are absorption and scattering attenuation coefficients, respectively. The unit of the overall attenuation coefficient, c(λ), is m${}^{-1}$. The channel loss can be represented as:

(2) $$h_{o}\left(d\right)=L\left(\lambda ,d\right)\frac{A_{r}\cos\theta \cos\varphi }{2\pi d^{2}},$$

where $A_{r}$ is the size of the photodetector (PD) in the receiver. θ and φ are the irradiation and incident angles between the LED and the user. *d* is the propagation distance between the LED and the user. L(λ,d) represents the link attenuation, which is calculated as:

(3) L(λ,d)=exp(−c(λ)·d).

To simplify the problem, we assume that the propagation distance and attenuation of absorption and scattering mainly impact the channel loss.

### 2.3. Channel Estimation Error and Pointing Error

**Figure 2:**
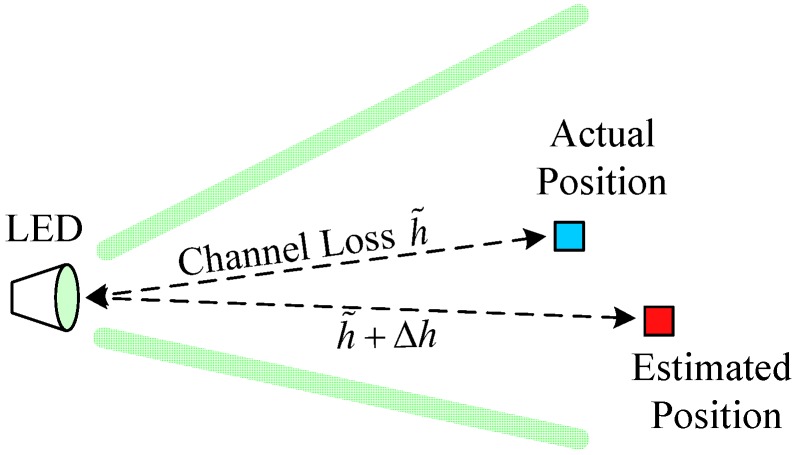
Pointing error caused by the fluctuation of transmitters and receivers.

In this paper, we take the channel estimation and pointing errors into account. Since the transmitters and receivers are fluctuant in the underwater environment, the pointing errors can result in the channel estimation error. As shown in Figure 2, the actual position of the receiver varies over time around the estimated location; therefore, due to the central limit theorem (CLT), we can model the channel uncertainty, Δh, caused by pointing error as a Gaussian distributed variable. Thus, the overall channel loss can be modeled as $h=\tilde{h}h_{o}\left(d\right)+\Delta h$.

Considering a turbulence scenario, $\tilde{h}$ represents the fading coefficient of the channel loss, which can be modeled with the log-normal distribution as [29]:

(4) $$f_{\tilde{h}}\left(\tilde{h}\right)=\frac{1}{\tilde{h}\sqrt{2\pi \sigma_{x}^{2}}}\exp\left(\frac{-{\left(\ln\left(\tilde{h}\right)-\mu_{x}\right)}^{2}}{8\sigma_{x}^{2}}\right),$$

where $\mu_{x}$ and $\sigma_{x}^{2}$ are the mean and variance of the log-normal distribution factor $x=\frac{1}{2}\ln\left(h\right)$ [30]. In this paper, we assume the pointing error is independent between LEDs and users, and this uncertainty can be modeled as a Gaussian random process with zero mean and variance $\sigma_{h}^{2}$.

### 2.4. Power and Bandwidth Constraints of LEDs

LEDs working as lighting sources and transmitters are nonlinear and power-limited devices with a bandwidth constraint.

**Figure 3:**
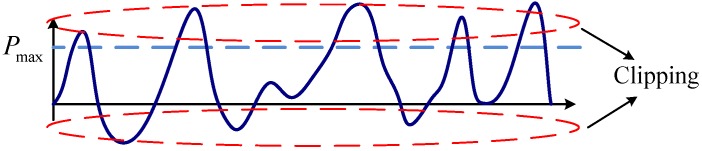
Illustration of zero and peak power limits for bipolar signals.

Since the optical OFDM signals can be modeled as Gaussian random variables, the magnitude can be very large. Due to the peak transmitted power constraint of LEDs, the high-valued signals greater than this power limit must be clipped. As shown in Figure 3, the signals beyond the peak power and below zero must be hard clipped. This clipping distortion is unrecoverable and can severely degrade the OFDM system performance.

Due to the slow rise-time of the LED, its bandwidth is limited, which impacts the data throughput of UOWC systems. In this paper, we compare DCO-, ACO-, and U-OFDM in UOWC systems with considerations of zero and peak power constraints, as well as the bandwidth limit.

## 3. Analysis of Optical OFDM for Underwater Communications

This section describes and analyzes the principles and performances of DCO-, ACO-, and U-OFDM in underwater communication systems.

### 3.1. DCO-OFDM

**Figure 4:**
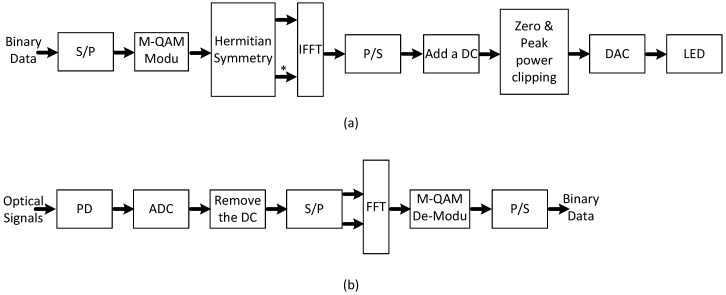
Block diagram of DCO-OFDM. (**a**) Structure of the transmitter; (**b**) structure of receiver [33]. P, parallel; S, serial; M-QAM, *M*-ary quadrature amplitude modulation.

DCO-OFDM is a basic method of optical OFDM and is widely used due to its low complexity [31,32]. Since only the non-negative signals can be transmitted, DCO-OFDM signals are derived by adding a DC bias to the bipolar OFDM signals. Figure 4 shows the block diagram of DCO-OFDM. In this figure, Parts (a) and (b) illustrate the transmitter and receiver structures, respectively. Hermitian symmetry is used to generate real signals for optical wireless communication systems at the transmitter. However, considering the peak power limit, the added DC bias cannot totally avoid zero clipping. In the meantime, adding the DC can introduce peak power clipping distortion. At the receiver, the DC bias needs to be removed first, then the M-QAM data can be recovered and demodulated.

In Figure 4a, after the inverse fast Fourier transform (IFFT), the discrete data for the kth subcarrier component, x[k], can be represented as:

(5) $$x\left[k\right]=\sum_{i=0}^{N-1}X_{i}\exp\left(\frac{j2\pi ki}{N}\right),\;\forall \;k=0,1,\cdots ,N-1,$$

where *N* represents the number of subcarriers used for modulation. $X_{i}$ is the data after M-QAM mapping for the ith subcarrier. Due to the Hermitian symmetry, we set $X_{N-1-i}=X_{i}^{*}$, ∀i=0,1,⋯,N−1. After the parallel to serial (P/S) converter, the mth sample of one DCO-OFDM can be represented as:

(6) $$x_{D}\left[m\right]=\beta \sum_{k=0}^{N-1}x\left[k\right]\delta \left[m-k\right]+b_{\mathrm{DC}},$$

where $b_{\mathrm{DC}}$ is the additive DC bias and β is a coefficient that controls the scale of the DCO-OFDM signals. Since we assume that the number of subcarriers, *N*, is usually very large (at least 64), $x_{D}\left[m\right]$ can be modeled as a Gaussian random variable with $b_{\mathrm{DC}}$ mean and $\sigma_{x}^{2}$ variance. Considering the zero and peak power clipping, the probability density function (pdf) of the DCO-OFDM signal can be represented as:

(7) $$\begin{array}{cc} f_{D}\left(x,b_{\mathrm{DC}}\right)= & \frac{1}{\sqrt{2\pi \sigma_{x}^{2}}}\exp\left(\frac{-{\left(x-b_{\mathrm{DC}}\right)}^{2}}{2\sigma_{x}^{2}}\right)\Pi \left(x,P_{m}\right)\\ & +\frac{1}{2}\mathrm{erfc}\left(\frac{b_{\mathrm{DC}}}{\sqrt{2\sigma_{x}^{2}}}\right)\delta \left(x\right)\\ & +\frac{1}{2}\mathrm{erfc}\left(\frac{P_{m}-b_{\mathrm{DC}}}{\sqrt{2\sigma_{x}^{2}}}\right)\delta \left(x-P_{m}\right), \end{array}$$

where $\Pi \left(x,P_{m}\right)=u\left(x\right)-u\left(x-P_{m}\right)$ is defined as a rectangular function and u(x) is the unit step function. erfc(x) is the complementary error function, which is defined as:

(8) $$\mathrm{erfc}\left(x\right)=\frac{2}{\sqrt{\pi }}\int_{x}^{\infty }\exp\left(-y^{2}\right)dy.$$

**Figure 5:**
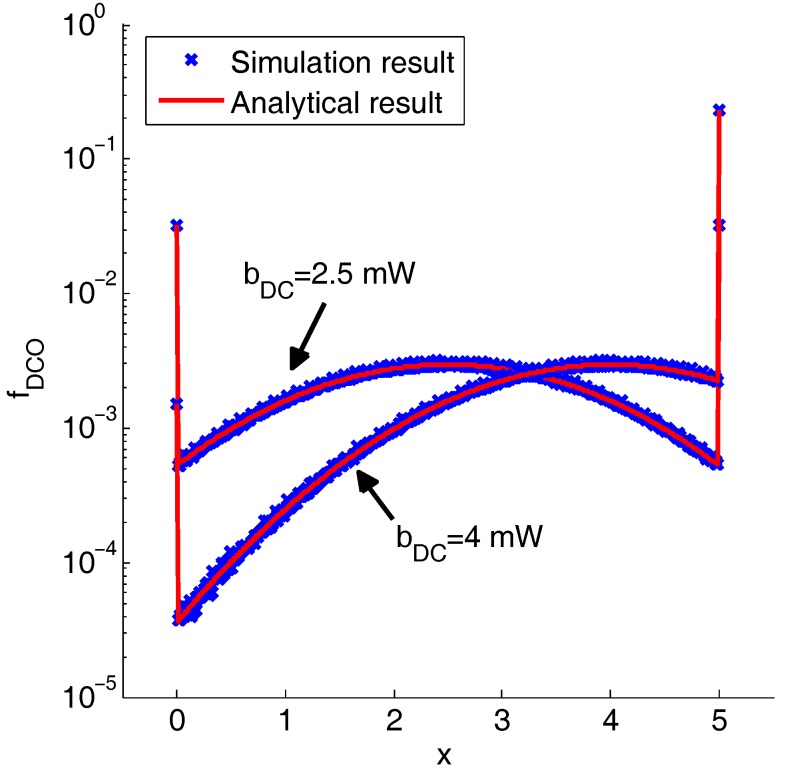
Simulation and analytical results of the pdf of the DCO-OFDM signal, β=20 and $P_{m}=5$ mW.

Figure 5 shows the pdf of the DCO-OFDM signal numerically. In this paper, simulation and analytical results are perfectly fit. From the results, zero clipping distortion is smaller than the peak clipping distortion when the DC bias is greater than half of the peak transmitted power. Otherwise, zero clipping distortion is greater than the peak clipping distortion.

Since the DCO-OFDM signal is modeled as a Gaussian random variable and the LED works as a nonlinear device, the sampled received signal then can be modeled based on the Bussgang theorem as [26,34]:

(9) $$\begin{array}{cc} y_{\mathrm{DCO}}\left[m\right] & =rh\left(\alpha_{D}\left(b_{\mathrm{DC}}\right)\cdot x_{D}\left[m\right]+c_{D}\left[m\right]\right)+n\left[m\right],\\ & =r\left(\tilde{h}h_{o}+\Delta h\right)\left(\alpha_{D}\left(b_{\mathrm{DC}}\right)\cdot x_{D}\left[m\right]+c_{D}\left[m\right]\right)+n\left[m\right] \end{array}$$

where *r* is the optical power to current converting rate. $\alpha_{D}\left(b_{\mathrm{DC}}\right)$ represents the clipping coefficient, which is a function of the DC bias, $b_{\mathrm{DC}}$. The calculation of $\alpha_{D}\left(b_{\mathrm{DC}}\right)$ is derived in the Appendix A, the result of which is shown as:

(10) $$\alpha_{D}\left(b_{\mathrm{DC}}\right)=1-\frac{1}{2}\left(\mathrm{erfc}\left(\frac{-P_{m}+b_{\mathrm{DC}}}{\sqrt{2\sigma_{x}^{2}}}\right)+\mathrm{erfc}\left(\frac{b_{\mathrm{DC}}}{\sqrt{2\sigma_{x}^{2}}}\right)\right).$$

In Equation (9), $c_{D}\left[m\right]$ is clipping noise, and n[m] is the sum of shot noise and thermal noise. $c_{D}\left[m\right]$ and n[m] are both modeled as Gaussian random variables with zero mean. The variance of the clipping noise can be calculated by:

(11) $$\begin{array}{cc} \sigma_{D}^{2} & =\int_{-\infty }^{0}x^{2}\cdot f_{D}\left(x\right)dx+\int_{P_{m}}^{\infty }{\left(x-P_{m}\right)}^{2}\cdot f_{D}\left(x\right)dx\\ & =\sqrt{\frac{\sigma_{x}^{2}}{8\pi }}\left(b_{\mathrm{DC}}-P_{m}\right)\exp\left(\frac{-{\left(b_{\mathrm{DC}}-P_{m}\right)}^{2}}{2\sigma_{x}^{2}}\right)\\ & -\sqrt{\frac{\sigma_{x}^{2}}{8\pi }}b_{\mathrm{DC}}\exp\left(\frac{-b_{\mathrm{DC}}^{2}}{2\sigma_{x}^{2}}\right)+\frac{1}{2}\left(\sigma_{x}^{2}+{\left(b_{\mathrm{DC}}-P_{m}\right)}^{2}\right)\\ & +\frac{1}{4}\mathrm{erfc}\left(\frac{b_{\mathrm{DC}}}{\sqrt{2\sigma_{x}^{2}}}\right)-\frac{1}{4}\mathrm{erfc}\left(\frac{b_{\mathrm{DC}}-P_{m}}{\sqrt{2\sigma_{x}^{2}}}\right) \end{array}$$

and the variance of n[m] is:

(12) $$\sigma_{n}^{2}=N_{o}R_{s}+2qr\bar{P}R_{s},$$

where $N_{o}$ and $R_{s}$ are thermal noise spectral density and symbol rate, respectively. *q* and $\bar{P}$ are the electronic charge and average received optical power, respectively. $\bar{P}$ is derived in the Appendix A. Therefore, the signal to noise ratio (SNR) using DCO-OFDM after FFT in the receiver, as shown in Figure 4b, can be calculated as:

(13) $$\gamma_{D}\left(d,\beta ,b_{\mathrm{DC}}\right)=\frac{{\left(\tilde{h}h_{o}\left(d\right)\beta \alpha_{D}\left(b_{\mathrm{DC}}\right)\right)}^{2}\sigma_{x}^{2}}{N(\left({\tilde{h}}^{2}h_{o}^{2}\left(d\right)+\sigma_{h}^{2}\right)\sigma_{D}^{2}+\sigma_{n}^{2})},$$

which is a function of β, *d*, and the DC bias. Considering the fading underwater channel, the average SNR can be calculated as:

(14) $$\begin{array}{cc} {\bar{\gamma }}_{D}\left(d,\beta ,b_{\mathrm{DC}}\right) & =\int_{0}^{\infty }\gamma_{D}\left(d,\beta ,b_{\mathrm{DC}}\right)f_{\tilde{h}}\left(\tilde{h}\right)d\tilde{h} \end{array}$$

Since the propagation distance is a concern in underwater optical wireless communication systems, we can obtain the optimal transmission distance, $d^{*}$, by optimizing the coefficient, β. The optimal transmission distance can be found by:

(15) $$\begin{array}{cc} d^{*} & =\max_{\beta ,b_{\mathrm{DC}}}\;d\\ s.t.\; & \;\mathrm{BER}\left(d,\beta ,b_{\mathrm{DC}}\right)<B^{\max}, \end{array}$$

where $B^{\max}$ is the allowed maximum bit error rate (BER) and BER(d,β) represents the BER using M-QAM given by a transmission distance and β, which approximates as:

(16) $$\mathrm{BER}\left(d,\beta ,b_{\mathrm{DC}}\right)\;\;\approx \;\;\frac{\sqrt{M}\;-\;1}{\sqrt{M}\;\log_{2}\;\left(\;\sqrt{M}\right)}\mathrm{erfc}\;\left(\;\sqrt{\frac{3{\bar{\gamma }}_{D}\left(d,\beta ,b_{\mathrm{DC}}\right)}{2(M-1)}}\;\right),$$

where *M* is the modulation constellation size for M-QAM.

### 3.2. ACO-OFDM

**Figure 6:**
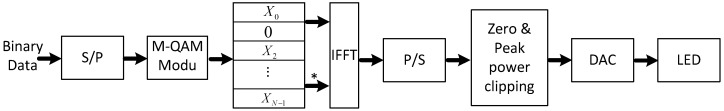
Block diagram of the ACO-OFDM transmitter [35].

ACO-OFDM is proposed for optical wireless communications, which can totally avoid the zero clipping distortion [22]. By using only the odd frequency subcarriers modulating data and leaving the even subcarriers blank, a bipolar signal is generated, as shown in Figure 6. Although zero clipping affects the ACO-OFDM signal, the interference caused by the zero clipping only happens on the even subcarriers. Therefore, no extra DC bias is needed. However, due to the zero clipping, the power of the data modulated using ACO-OFDM is reduced by half [22].

Similar to the DCO-OFDM, we can derive the pdf of the ACO-OFDM signal, which is represented as:

(17) $$\begin{array}{cc} f_{A}\left(x\right)= & \frac{1}{\sqrt{\pi \sigma_{x}^{2}}}\exp\left(\frac{-x^{2}}{\sigma_{x}^{2}}\right)\Pi \left(x,P_{m}\right)\\ & +\frac{1}{2}\delta \left(x\right)+\frac{1}{2}\mathrm{erfc}\left(\frac{P_{m}}{\sqrt{\sigma_{x}^{2}}}\right)\delta \left(x-P_{m}\right). \end{array}$$

**Figure 7:**
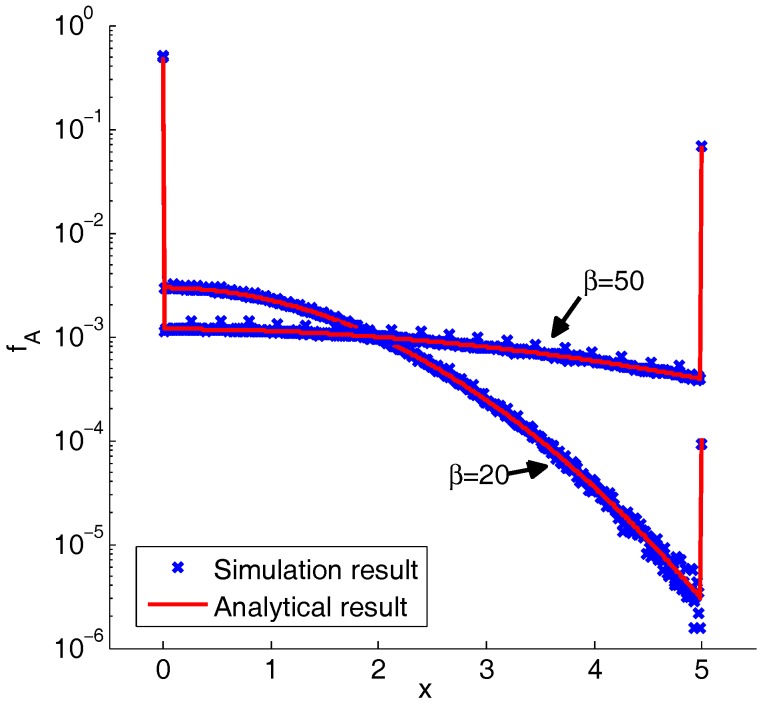
Simulation and analytical results of the pdf of the ACO-OFDM signal.

Figure 7 shows the simulation and analytical results of the ACO-OFDM signal’s pdf. From the results, a larger β can introduce more peak power clipped distortions, as well as a higher SNR. Thus, there is a compromise of β. Based on the Bussgang theorem, the clipping coefficient of ACO-OFDM can be obtained by:

(18) $$\alpha_{A}=\frac{1}{2}-\frac{1}{2}\mathrm{erfc}\left(\frac{P_{m}}{\sqrt{2\sigma_{x}^{2}}}\right).$$

Due to the peak transmitted power constraint, the variance of clipping noise of ACO-OFDM can be calculated by:

(19) $$\begin{array}{cc} \sigma_{A}^{2} & =\int_{P_{m}}^{\infty }{\left(x-P_{m}\right)}^{2}\cdot f_{A}\left(x\right)dx\\ & =\frac{1}{2}\left(P_{m}^{2}+\sigma_{x}^{2}\right)\mathrm{erfc}\left(\frac{P_{m}^{2}}{2\sigma_{x}^{2}}\right)-\frac{1}{2}\sigma_{x}P_{m}\exp\left(\frac{-P_{m}^{2}}{2\sigma_{x}^{2}}\right). \end{array}$$

The received SNR using ACO-OFDM can be calculated by:

(20) $$\gamma_{A}\left(d,\beta \right)=\frac{{\left(\tilde{h}h_{o}\left(d\right)\beta \alpha_{A}\right)}^{2}\sigma_{x}^{2}}{2N(\left({\tilde{h}}^{2}h_{o}^{2}\left(d\right)+\sigma_{h}^{2}\right)\sigma_{A}^{2}+\sigma_{n}^{2})}.$$

Then, the average SNR can be calculated by:

(21) $${\bar{\gamma }}_{A}\left(d,\beta \right)=\int_{0}^{\infty }\gamma_{A}\left(d,\beta \right)f_{\tilde{h}}\left(\tilde{h}\right)d\tilde{h}$$

### 3.3. U-OFDM

The unipolar OFDM works as a novel optical OFDM technique, which was proposed in [21]. It has advantages over DCO- and ACO-OFDM. Unlike ACO-OFDM, all the subcarriers can be used for modulating. Being superior to DCO-OFDM, there is no zero clipping for U-OFDM.

**Figure 8:**
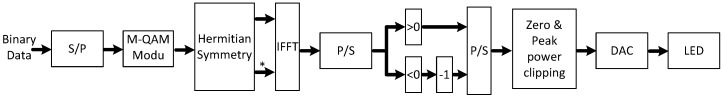
Block diagram of the unipolar (U)-OFDM transmitter [21].

U-OFDM successively transmits the positive and negative information using two time slots to avoid the zero clipping distortions. A block diagram of U-OFDM is shown in Figure 8. When transmitting the positive values of signals, zeros are set at the negative values’ positions. Then, the absolute value of the negative information is sent. To recover the data easily, the two time slots should have the same length. For example, if the bipolar information is [1,−2,−4,2,−5,3,0,−1], the U-OFDM transmits [1,0,0,2,0,3,0,0] using the first slot. Then, [0,2,4,0,5,0,0,1] are transmitted in the following slot. Thus, only the peak power constraint can affect the signal. At the receiver, the signal received from the first and second slots can be used to reconstruct the original bipolar signal. Obviously, U-OFDM requires a twice larger bandwidth than DCO-OFDM when transmitting the same bit rate using the equivalent modulation scheme.

Similar to DCO- and ACO-OFDM, we can calculate the clipping coefficient, clipping noise, and SNR for U-OFDM. After computing, the clipping coefficient for U-OFDM can be represented as:

(22) $$\alpha_{U}=1-\mathrm{erfc}\left(\frac{P_{m}}{\sqrt{2\sigma_{x}^{2}}}\right).$$

Since U-OFDM signal is unipolar, the pdf can be represented as:

(23) $$\begin{array}{cc} f_{U}\left(x\right)= & \frac{1}{\sqrt{2\pi \sigma_{x}^{2}}}\exp\left(\frac{-x^{2}}{2\sigma_{x}^{2}}\right)\Pi \left(x,P_{m}\right)\\ & +\frac{1}{2}\delta \left(x\right)+\frac{1}{2}\mathrm{erfc}\left(\frac{P_{m}}{\sqrt{2\sigma_{x}^{2}}}\right)\delta \left(x-P_{m}\right). \end{array}$$

Thus, the variance of clipping noise can be calculated and obtained as:

(24) $$\begin{array}{cc} \sigma_{U}^{2} & =\int_{P_{m}}^{\infty }{\left(x-P_{m}\right)}^{2}\cdot f_{U}\left(x\right)dx\\ & =\left(P_{m}^{2}+\sigma_{x}^{2}\right)\mathrm{erfc}\left(\frac{P_{m}^{2}}{2\sigma_{x}^{2}}\right)-\sigma_{x}P_{m}\exp\left(\frac{-P_{m}^{2}}{2\sigma_{x}^{2}}\right). \end{array}$$

The received SNR of U-OFDM can be represented as:

(25) $$\gamma_{U}\left(d,\beta \right)=\frac{{\left(\tilde{h}h_{o}^{2}\beta \alpha_{U}\right)}^{2}\sigma_{x}^{2}}{2N(\left({\tilde{h}}^{2}h_{o}^{2}+\sigma_{h}^{2}\right)\sigma_{U}^{2}+\sigma_{n}^{2})},$$

and the average SNR for U-OFDM is calculated by:

(26) $${\bar{\gamma }}_{U}\left(d,\beta \right)=\int_{0}^{\infty }\gamma_{U}\left(d,\beta \right)f_{\tilde{h}}\left(\tilde{h}\right)d\tilde{h}.$$

Since U-OFDM transmits positive and negative signals successfully, after reconstructing the signal at the receiver, the additive Gaussian noise variance is twice that of ACO- or DCO-OFDM.

### 3.4. Modulation Index Optimization

The modulation index, β, in DCO-, ACO-, and U-OFDM can be optimized to improve the SINR. Since β controls the scale of the OFDM signals, a larger β can provide a higher signal power. However, the larger scale of the OFDM signal can introduce more clipping distortion, which is caused by the high peak to average power ratio of the OFDM signal and the peak transmitted power constraint of the LED. Therefore, the modulation index needs to be optimized to trade off the signal power and the clipping distortion. To achieve a high data rate, the modulation constellation size can be adaptively adjusted based on the SINR for a given β.

## 4. Band-Limited Channel

In this section, we discuss and analyze the effects of the band-limited underwater optical channel. Due to the scattering and multi-reflection effects of the underwater environment, the channel is fading and frequency selective. Since LEDs are used as transmitters in the underwater optical wireless communication systems, the band-limited LED dominates the overall bandwidth of the systems. Usually, the 3-dB bandwidth of a white-color LED is limited only to a few tens of MHz. Thus, we assume that the overall bandwidth of the underwater optical wireless communication system is round 15 MHz [36].

**Figure 9:**
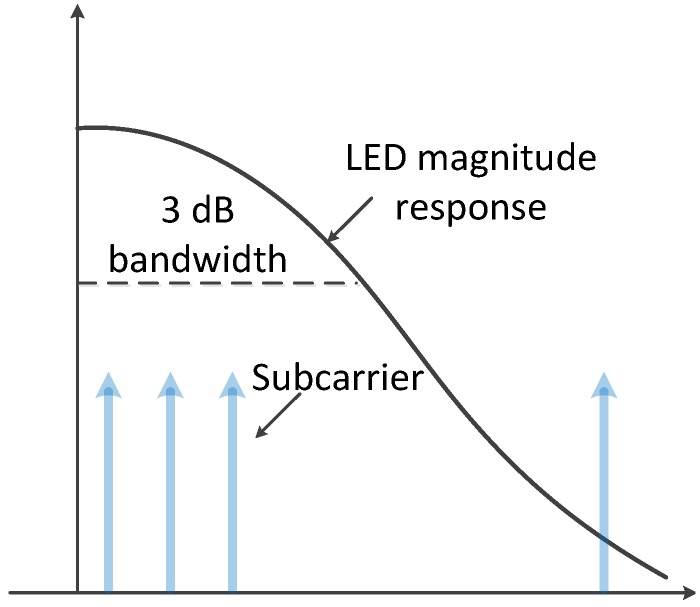
An illustration of the LED magnitude response.

**Figure 10:**
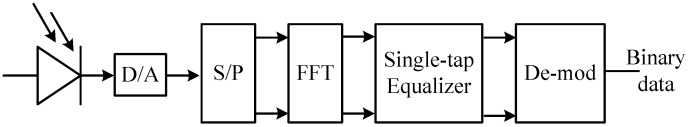
A block diagram of the optical OFDM receiver with a single-tap equalizer.

As shown in Figure 9, the LED can be modeled as a first order low-pass filter. Due to the low-pass characteristics, the SNR for each subcarrier is different. In addition, the phase distortion introduced by the band-limited channel can affect the modulated M-QAM data. Thus, a single-tap equalizer and a bitloading algorithm in the frequency domain can be applied to correct the phase distortion and maximize the total throughput. We assume that the channel response can be perfectly estimated; thus, the phase distortion can be totally eliminated. As shown in Figure 10, the single-tap equalizer is set after the FFT. Using a bitloading algorithm, the channel spectrum can be efficiently utilized.

## 5. Numerical Results

In this section, numerical results of the comparison of the DCO-, ACO-, and U-OFDM techniques for UOWC systems are shown. The parameters used to obtain the numerical results are shown in Table 3. This is a baseline for all the numerical results in this paper. Since the attenuation coefficient varies over temperature, concentration of salt, and spectrum of transmitted light, to simplify the problem, we assume the pure sea water condition is used [4]. The light with a wavelength from 480 nm–520 nm is used, which has the lowest absorption rate in sea water. We also assume that the thermal noise is dominated. For the photodetector (PD), a lens can be integrated to increases the effective area and collect more power. For the numerical results, the bandwidth limitation is taken into account. We mainly consider the bandwidth limitation from LEDs since the PD’s bandwidth can be much broader compared with the symbol rate tested in this paper [37,38]. The numerical results illustrated in this paper are based on the theory discussed above. We have tested and verified the theoretical results by using simulation. The forward error control (FEC) codes need to be applied in a practical situation for a better BER performance, which does not change the relative results among DCO-, ACO-, and U-OFDM obtained in this paper. Therefore, there is no need to consider the advantages of FEC in this paper. In addition, we set a $10^{-3}$ BER as the constraint when maximizing the bit rate, since, with the help of FEC, the BER can be easily and significantly improved.

**Figure 11:**
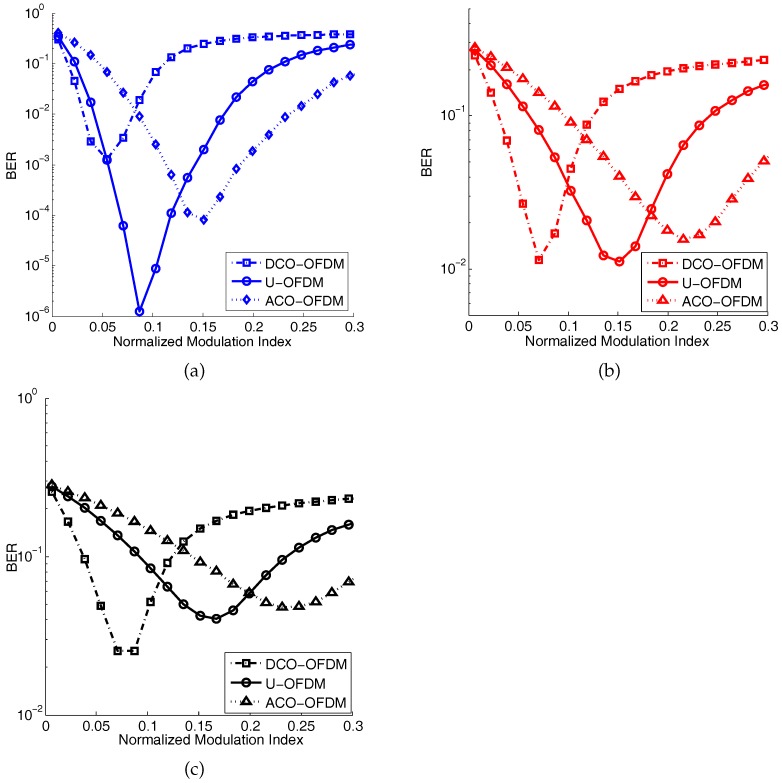
Average BER comparison of DCO-, ACO-, and U-OFDM with $R_{s}=30$ Msps and 64-QAM. Propagation distance is 1 m. (**a**) Ideal channel with no bandwidth limit; (**b**) the 3-dB bandwidth of the channel is 25 MHz; (**c**) the 3-dB bandwidth of the channel is 10 MHz.

The numerical results of BER using Equation (16) are shown in Figure 11. In this figure, the normalized modulation index is used, which is $\beta /N/P_{m}$. Figure 11a–c illustrates the average BER of the optical OFDM tested under different channel conditions, respectively. In general, an increasing modulation index first leads to a better BER performance for all the tested optical OFDM. Then, continued increasing of the modulation index can introduce more clipping distortion caused by the peak power constraint; therefore, the BER worsens. Since an increasing modulation index improves the SNR, yet introduces more clipping distortion, an optimal modulation index needs to be found.

The ideal channel (no bandwidth constraint) case is shown in Figure 11a. Due to the less nonlinear distortion and higher power efficiency of U-OFDM compared with DCO- and ACO-OFDM, the BER performance of U-OFDM is better than DCO- and ACO-OFDM. Considering the channel bandwidth constraint, the BER performances of U- and ACO-OFDM become worse than DCO-OFDM. When the 3-dB bandwidth of the channel is 25 MHz, as shown in Figure 11b, the minimum BER of U-OFDM is similar to DCO-OFDM. For a more narrow bandwidth case (3 dB bandwidth is 10 MHz), as shown in Figure 11c, the minimum BER of U-OFDM is higher than that of DCO-OFDM since U-OFDM requires a wider bandwidth than DCO-OFDM when transmitting the same data rate. ACO-OFDM is even worse than U-OFDM due to its lower bandwidth utilization efficiency. In summary, due to different channel bandwidths, the best optical OFDM scheme can be selected to obtain a better BER performance.

**Figure 12:**
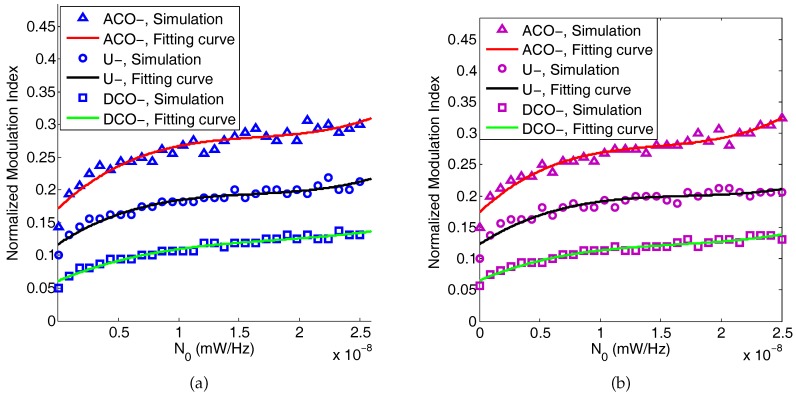
Normalized modulation index of the minimum BER for different noise levels. (**a**) The 3-dB bandwidth of the channel is 25 MHz; (**b**) the 3-dB bandwidth of the channel is 10 MHz.

How to choose the optimal modulation index for DCO-, ACO-, and U-OFDM is a problem that needs to be discussed. Figure 12 shows the simulation results of the optimal normalized modulation index for different noise spectral density. In this result, the transmitted peak power and the data rate are fixed. Figure 12a,b shows the relationship between the modulation index and the noise level for two different band-limited channels, respectively. From the simulation results, we use cubic equations to fit and model the relationship between the optimal normalized modulation index and noise spectral density based on the simulation results. Comparing (a) and (b) in Figure 12, the two different bandwidths illustrate a similar phenomenon. The fitting equations for DCO-, U-, and ACO-OFDM are represented as:

(27) $$\begin{array}{cc} \mathrm{DCO}-\mathrm{OFDM}: & \frac{\beta }{NP_{\max}}=6.7\times 10^{21}\times N_{0}^{3}-3.6\times 10^{14}\times N_{0}^{2}+7.8\times 10^{6}\times N_{0}+0.061\\ U-\mathrm{OFDM}: & \frac{\beta }{NP_{\max}}=1.3\times 10^{22}\times N_{0}^{3}-6.9\times 10^{14}\times N_{0}^{2}+1.2\times 10^{7}\times N_{0}+0.12\\ \mathrm{ACO}-\mathrm{OFDM}: & \frac{\beta }{NP_{\max}}=2.5\times 10^{22}\times N_{0}^{3}-1.1\times 10^{15}\times N_{0}^{2}+1.8\times 10^{7}\times N_{0}+0.17, \end{array}$$

where these equations can be used to predict the optimal modulation index for DCO-, ACO-, and U-OFDM in noisy environments with different noise levels.

**Figure 13:**
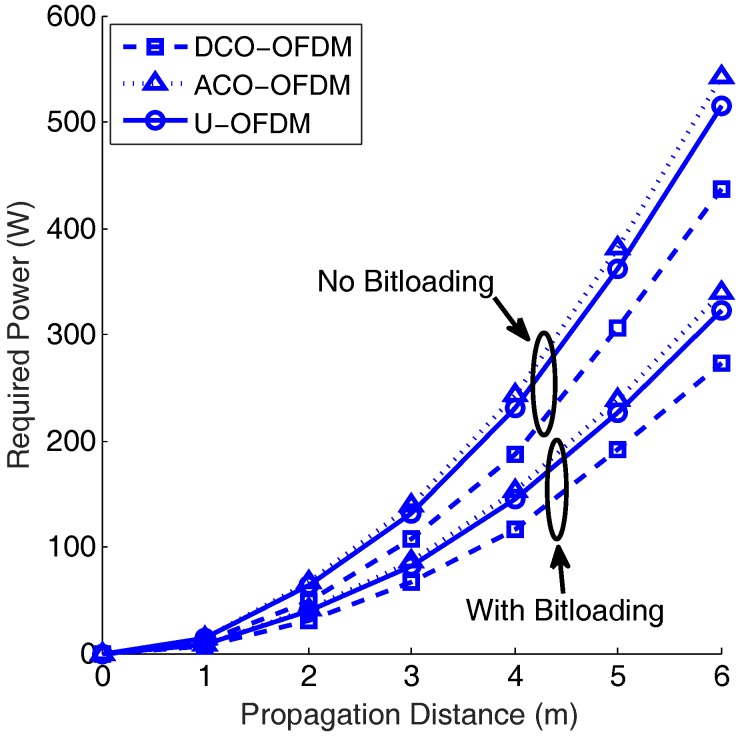
Required transmitted power for different propagation distances for a $10^{-3}$ BER. The 3-dB bandwidth is 10 MHz. The bit rate is 100 Mbps.

Figure 13 shows analytical results of the required power using DCO-, ACO-, and U-OFDM for different propagation distances with a 10-MHz, 3-dB bandwidth. In this figure, the cases with and without the bitloading algorithm are compared. Since the bandwidth tested is narrow, DCO-OFDM has a better performance than ACO- and U-OFDM, which is consistent, as shown in Figure 11. Without using the bitloading algorithm, the required power of the three optical OFDM schemes tested is about 30% higher than the case with bitloading. Since a single LED can only provide about tens of Watts [39,40], a LED-array with multiple LEDs needs to be used when a high power is required.

**Figure 14:**
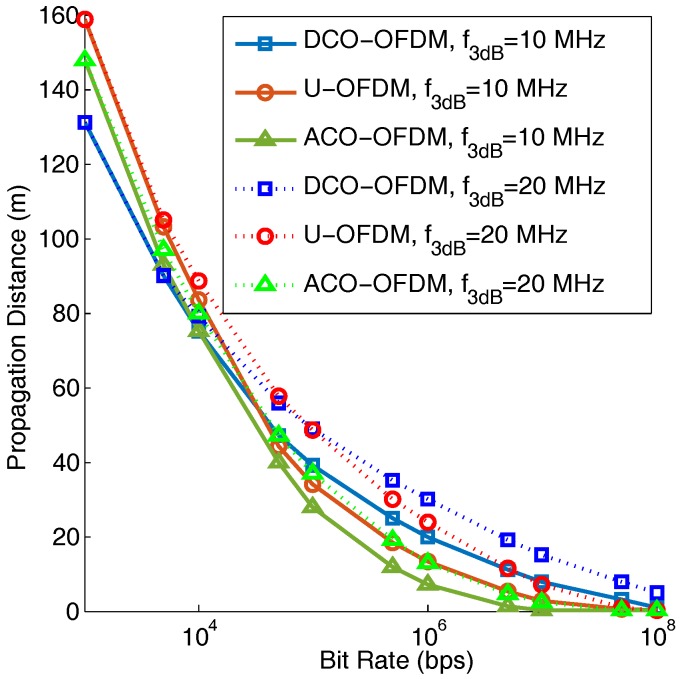
The maximum propagation distance for different bit rates. Peak transmitted power is fixed, and a bitloading algorithm is used.

Figure 14 shows the maximum propagation distance achieved using different transmission bit rates through a band-limited channel. In this result, a bitloading algorithm is used [41]. When the symbol rate is low compared to the channel bandwidth, U-OFDM can support a longer propagation distance than ACO- and DCO-OFDM due to its higher power efficiency and less nonlinear distortion than ACO- and DCO-OFDM. For the higher bit rates case, DCO-OFDM outperforms ACO- and U-OFDM due to its higher spectral efficiency. Depending on different required bit rates and channel bandwidth, we can choose the proper optical OFDM technique to obtain a good propagation and BER performance.

**Figure 15:**
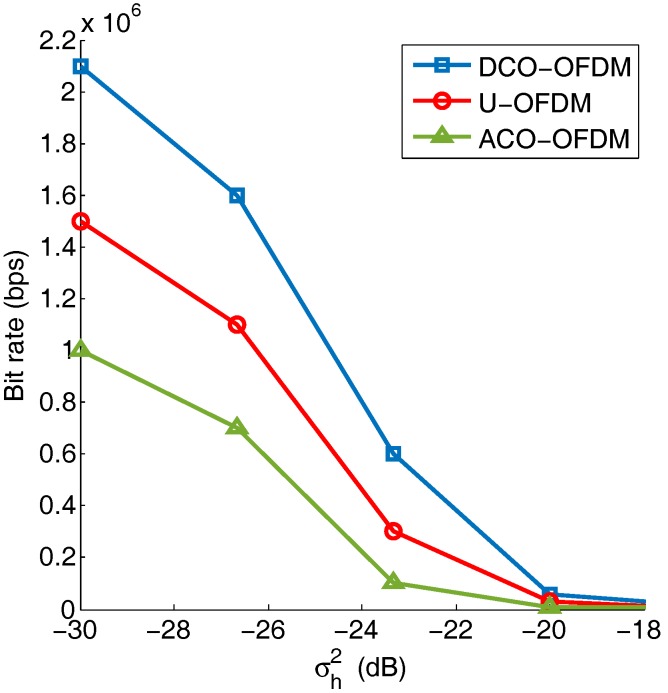
The maximum bit rate for DCO-, ACO-, and U-OFDM with an increasing $\sigma_{h}^{2}$. The propagation distance is 20 m, and the 3-dB bandwidth of the LED is 10 MHz.

A bit rate comparison of DCO-, ACO-, and U-OFDM in underwater optical wireless communication systems is shown in Figure 15. In this figure, the channel estimation error caused by pointing error is considered, and the bitloading algorithm is used to improve the throughput. In general, the maximum data rate for all the techniques tested drops as the channel uncertainty variance, $\sigma_{h}^{2}$, increases. Based on the theoretical analysis, the SNR dramatically decreases for an increasing channel uncertainty variance. For this result, since a severely-limited LED bandwidth is used, DCO-OFDM outperforms ACO- and U-OFDM, which is consistent with the previous analysis and results.

## 6. Conclusions

In this paper, we compare, discuss, and analyze DCO-, ACO-, and U-OFDM in underwater optical communication systems. Since the peak transmitted power is limited, distortions caused by the peak power are taken into account. We propose to optimize the modulation index and compromise the clipping effects and SNR. By optimizing the SNR, the achievable transmission distance is obtained by the given transmission bit rate and BER performance. Considering the bandwidth limitation, we find the relationship between the optimal modulation index and the noise spectral density, which can guide us to achieve the best BER performance in different noisy environments. For the channels with different bandwidths, we compare the performances of DCO-, ACO-, and U-OFDM in underwater OWC systems. When the required bit rate is high (compared to the bandwidth), DCO-OFDM can provide a longer propagation distance than ACO- and U-OFDM. Otherwise, U-OFDM outperforms ACO- and DCO-OFDM.

## 7. Future Work

In future work, an OFDM algorithm with peak-to-average-power-ratio (PAPR) reduction needs to be explored. Shot noise and the effects of different water conditions should be considered.

## Figures and Tables

**Table 1:** Notation list. DCO, DC-biased optical; ACO, asymmetrically-clipped optical.

a(λ):	absorption attenuation
b(λ):	scattering attenuation
α:	clipping coefficient
β:	modulation index
*h*:	channel loss
$A_{r}$:	size of the photodetector
*d*:	propagation distance
*N*:	Number of subcarriers for OFDM
$x_{D}\left[m\right]$	mth sample for the DCO-OFDM signal
$x_{U}\left[m\right]$	mth sample for the UCO-OFDM signal
$x_{A}\left[m\right]$	mth sample for the ACO-OFDM signal
$b_{\mathrm{DC}}$	DC bias for DCO-OFDM
$f_{D}$	probability density function of the DCO-OFDM signal
$f_{U}$	probability density function of the UCO-OFDM signal
$f_{A}$	probability density function of the ACO-OFDM signal
$\bar{P}$	average optical power
$P_{m}$	peak transmitted power

**Table 2:** Typical attenuation coefficients for different water types [27].

Water Types	Absorption (m${}^{-1}$)	Scattering (m${}^{-1}$)
Pure Sea Water	0.053	0.003
Clear Ocean	0.114	0.037
Coastal Ocean	0.179	0.219
Turbid Harbor	0.295	1.875

**Table 3:** Parameters used in this paper.

Responsivity	0.45 A/W
Equivalent area of the PD using a lens	$10^{-4}\;m^{2}$
Thermal noise density, $N_{0}$	$1.3\times 10^{-13}$ mW/Hz
Radiation optical power of each lamp, $P_{m}$	10 W
LED semi-angle	30°
a(λ)	0.053 ($m^{-1}$)
b(λ)	0.003 ($m^{-1}$)
c(λ)	0.056 ($m^{-1}$)
Number of subcarriers, *N*	128

## Appendix A

This Appendix gives the derivation of the clipping coefficient and the average optical power of the DCO-, ACO-, and U-OFDM signals.

(A1) $$\begin{array}{cc} \alpha_{D}\left(b_{\mathrm{DC}}\right) & =\frac{1}{\sigma_{x}^{3}\sqrt{2\pi }}\int_{-\infty }^{\infty }x\psi_{D}\left(x\right)\exp\left(\frac{-{\left(x-b_{\mathrm{DC}}\right)}^{2}}{2\sigma_{x}^{2}}\right)dx\\ & =\frac{1}{\sigma_{x}^{3}\sqrt{2\pi }}\left(\int_{0}^{P_{m}}x^{2}\exp\left(\frac{-{\left(x-b_{\mathrm{DC}}\right)}^{2}}{2\sigma_{x}^{2}}\right)dx\right.\\ & \left.+P_{m}\int_{P_{m}}^{\infty }x\exp\left(\frac{-{\left(x-b_{\mathrm{DC}}\right)}^{2}}{2\sigma_{x}^{2}}\right)dx\right)\\ & =1-\frac{1}{2}\left(\mathrm{erfc}\left(\frac{-P_{m}+b_{\mathrm{DC}}}{\sqrt{2\sigma_{x}^{2}}}\right)+\mathrm{erfc}\left(\frac{b_{\mathrm{DC}}}{\sqrt{2\sigma_{x}^{2}}}\right)\right). \end{array}$$

where:

(A2) $$\psi_{D}\left(x\right)\;=\;\left\{\begin{array}{c} 0,\;\;\;\;\;\;x<0\\ x,\;\;\;\;0<x<P_{m}\\ P_{m},\;\;\;\;x>P_{m}, \end{array}\right.$$

(A3) $$\begin{array}{cc} \alpha_{U} & =\frac{1}{\sigma_{x}^{3}\sqrt{2\pi }}\int_{-\infty }^{\infty }x\psi_{U}\left(x\right)\exp\left(\frac{-x^{2}}{2\sigma_{x}^{2}}\right)dx\\ & =\frac{1}{\sigma_{x}^{3}\sqrt{2\pi }}\left(-P_{m}\int_{-\infty }^{-P_{m}}x\exp\left(\frac{-x^{2}}{2\sigma_{x}^{2}}\right)\right.dx\\ & +\int_{-P_{m}}^{P_{m}}x^{2}\exp\left(\frac{-x^{2}}{2\sigma_{x}^{2}}\right)dx\\ & \left.+P_{m}\int_{P_{m}}^{\infty }x\exp\left(\frac{-x^{2}}{2\sigma_{x}^{2}}\right)dx\right)\\ & =1-\mathrm{erfc}\left(\frac{P_{m}}{\sqrt{2\sigma_{x}^{2}}}\right), \end{array}$$

where:

(A4) $$\psi_{U}\left(x\right)\;=\;\left\{\begin{array}{c} -P_{m},\;\;x<-P_{m}\\ x,\;\;\;\;-P_{m}<x<P_{m}\\ P_{m},\;\;\;\;x>P_{m}. \end{array}\right.$$

For DCO-OFDM, the average optical power can be calculated by:

(A5) $$\begin{array}{cc} \bar{P}\left(b_{\mathrm{DC}}\right) & =\int_{0}^{P_{m}}xf_{D}\left(x,b_{\mathrm{DC}}\right)dx\\ & =\int_{0}^{P_{m}}x\frac{1}{\sqrt{2\pi \sigma_{x}^{2}}}\exp\left(\frac{-{\left(x-b_{\mathrm{DC}}\right)}^{2}}{2\sigma_{x}^{2}}\right)dx\\ & +P_{m}\frac{1}{2}\mathrm{erfc}\left(\frac{P_{m}-b_{\mathrm{DC}}}{\sqrt{2\sigma_{x}^{2}}}\right)\\ & =\frac{b_{\mathrm{DC}}}{2}\left(\mathrm{erfc}\left(\frac{b_{\mathrm{DC}}}{\sqrt{2\sigma_{x}^{2}}}\right)-\mathrm{erfc}\left(\frac{b_{\mathrm{DC}}-P_{m}}{\sqrt{2\sigma_{x}^{2}}}\right)\right)\\ & +\frac{\sigma_{x}}{\sqrt{2\pi }}\left(\exp\left(\frac{-b_{\mathrm{DC}}^{2}}{2\sigma_{x}^{2}}\right)-\exp\left(\frac{-{\left(b_{\mathrm{DC}}-P_{m}\right)}^{2}}{2\sigma_{x}^{2}}\right)\right)\\ & +P_{m}\frac{1}{2}\mathrm{erfc}\left(\frac{P_{m}-b_{\mathrm{DC}}}{\sqrt{2\sigma_{x}^{2}}}\right) \end{array}$$
